# Is all movement equal at every age? Differentiating the impact of physical activity types on depressive symptoms and mental acuity in an aging India

**DOI:** 10.1186/s13690-026-01943-x

**Published:** 2026-05-11

**Authors:** Madhurima Sharma, Indrajit Goswami

**Affiliations:** 1 International Institute for Population Sciences, Mumbai, 400088 India; 2 Tata Institute of Social Sciences, Mumbai, 400088 India

**Keywords:** Physical activity, Cognitive decline, Depressive symptoms, Older adults, India

## Abstract

**Background:**

The geriatric population has been growing worldwide, leading to a higher prevalence of cognitive conditions in older persons, particularly in lower- and middle-income countries like India. Physical activity (PA) is associated with improved mental well-being and lessen these symptoms, but most of the researches are carried out on the Western nations and there is a gap in the Indian context.

**Objective:**

To examine the correlation amongst different forms of physical activities and their association with cognitive impairment (CoI) and depressive symptoms among Indian older adults.

**Methodology:**

We analysed dataset from the wave 1 of the Longitudinal Ageing Study in India (2017-18), covering 59,813 respondents aged 45 years or older. The Adjusted Prevalence Ratios (APRs) were assessed utilizing the Multivariable Poisson Regression models, accounting for demographic and socioeconomic factors.

**Findings:**

Estimated prevalence of CoI among older adults was 11.11%, while depressive symptoms affected 27.39% of the study population. Individuals engaging in vigorous physical activity had a lower prevalence of CoI (7.72%) than those who were not involved in such activities (12.27%) (APR of CoI: 1.07; 95% CI: 0.94–1.22). Holistic PA, such as yoga, was associated with the lowest prevalence of CoI (4.69%) compared to those not engaging in such activities (11.77%) with an APR of 1.42 (95% CI: 1.22–1.67). Depressive symptoms were less prevalent among individuals engaged in any form of physical activity. The lowest proportions were observed among participants involved in holistic activities (23.76%) compared to non-participants (27.76%).

**Conclusion:**

Engagement in physical activities, including holistic practices, can be beneficial in reducing the risk of cognitive function deterioration and depressive symptoms. Engagement in these activities can be promoted as a non-pharmaceutical therapeutic approach to alleviate the mental well-being and cognitive functioning in this population. More research evidence, especially from longitudinal studies, is needed. These correlations need to be verified and further substantiated through longitudinal research.

**Table:** 

Text box 1. Contributions to the literature
• This study attempts to explore the understudied correlation between PA and mental health among senior citizens in India, underscoring cultural and socioeconomic nuances.
• It suggests that holistic PA, such as yoga, reduce cognitive deterioration and depressive symptoms.
• This paper highlights the non-pharmacological interventions commonly aligned with India’s socio-cultural context.
• The findings call for, more substantial public health efforts to encourage physical activity among older adults, aiming to boost their cognitive and emotional well-being, an urgent priority for lower-and middle-income countries facing rapidly ageing populations.

## Introduction

The World Health Organization (WHO) has documented that the worldwide proportion of individuals aged 60 years and above projected to increase twofold, increasing from 12% in 2015 to 22% by 2050 [1]. The population of older individuals in the world is growing, and most them reside in Low- and middle-income countries such as India and China [2]. The phenomenon of population aging is accompanied by both physical and cognitive decline. India has 98 million senior citizens as of the 2011 Census; it is anticipated to increase to 143 million by 2021, with women comprising up 51% of the population [3]. Between 2002 and 2022, the average Indian’s life expectancy grew from 64.6 to 70.19 years [4]. By 2050, individuals aged ≥ 60 are predictable to make up 19.5% of India’s entire population a demographic shift that raises serious concerns and calls for prompt policy action [5].

As the years go by, the human brain naturally undergoes shifts that affect a wide range of mental abilities. Cognitive function encompasses memory and language, visuospatial skills, executive function, calculation, comprehension, and judgment [6]. As people age, many begin to experience some level of cognitive decline, which can make everyday life more difficult and lessen their overall sense of well-being, but also affects their ability to perform basic daily activities, shortens their remaining life expectancy, and increases their risk of death [7]. With advancing age mental disorders lead to psychological alterations and a reduction in emotional well-being [8]. Consequently, understanding the physiological and neurological mechanisms underlying age-related cognitive changes is crucial. Equally important is developing preventive strategies to mitigate their adverse effects in public health and clinical practice. Notably, the estimated volume of individuals suffering from dementia in India is close to 5.3 million, and this estimate is projected to increase threefold by 2050. This presents significant public health and socioeconomic concerns in the near future [9, 10].

Worldwide, depressive symptoms is a widespread mental disorder that severely affects millions of individuals, hindering their daily functioning and overall well-being [11–13]. It is characterized by prolonged periods of apathy, diminished motivation, and persistent feelings of desolation, which alter the overall sleep quality, appetite, and daily functioning, increase the risk of chronic diseases [14], cause fatigue, raise both suicide and non-suicide mortality rates [15], lack of concentration, lower self-esteem [16], and contribute to a high disease burden [17]. The situation is significantly worse among older adults, with 6–10% of adult seniors experiencing depression [18].

A recent systematic review study based on Indian population showed that, the estimated pooled prevalence of depression among older adults was 34.4%, with higher rates observed among females and those in rural areas [19]. Studies have found that despite the availability of treatment for depression, 75% of persons in lower-middle-income countries (LMICs) lack access to clinical intervention. This is due to factors such as inaccessibility of services, insufficient resources and personnel, and stigma surrounding mental health disorders [20]. In India, individuals aged 60 or above account for 10.1% of the entire population. If depressive symptoms among this age group goes undetected and untreated, it could significantly contribute to the overall disease burden [19].

Physical activity (PA) is crucial for preventing and controlling non-communicable diseases. Additionally, engaging in physical exercise endorses mental health by averting or postponing cognitive decline and depressive symptoms and anxiety, while also aiding in the maintenance of body mass and overall well-being [21]. There is an association between lower levels of PA and the incidences of depressive symptoms and anxiety in older persons [22]. A growing body of evidence have consistently indicated that physical activity can positively impact the cognition of older adults [23–25]. For instance, Fratiglioni et al. found that three modifiable lifestyle factors participating in cognitively stimulating leisure activities, maintaining an active social network, and regularly exercising can substantially slow cognitive decline and reduce the risk of developing dementia as age advances [26]. A meta-analysis of fifteen cohort based studies with longitudinal follow-up periods from 1 to 12 years and persons not suffering from dementia discovered a significant inverse relationship between physical exercise and cognitive deterioration [27]. Therefore, PA is recognized as an efficacious preventative intervention against depressive symptoms and for improving overall mental health. One of the primary objectives of public health is to promote healthy living by encouraging positive lifestyle changes through health-enhancing physical activity (HEPA) [28].

**Fig. 1:**
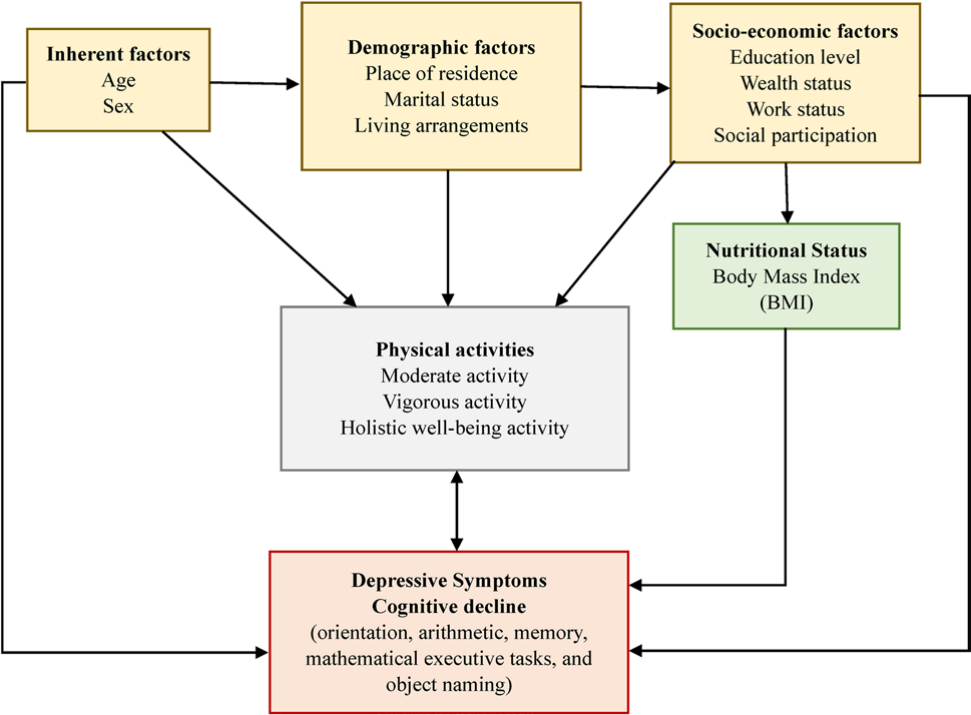
Conceptual framework for depressive symptoms and cognitive decline among older adults

Remarkably, most studies investigative the association between PA and symptoms of depression, as well as CoI, have largely been carried out in Western nations [29–32]. There is still a significant research gap in comprehending the relationship between PA and depressive symptoms and cognitive functioning in older persons in the Indian context. In a cultural context with limited resources, where lifestyle practices like diet and types of PA differ from those in affluent nations, the association between PA, depressive symptoms, and cognitive wellbeing is likely to differ. Grounded on the existing evidences and contextual background, we hypothesize that lower engagement in PA is associated with a higher likelihood of depressive symptoms and cognitive decline amongst older Indian adults. To address this evidentiary gap, the current study systematically investigated the associations between PAs and depressive symptoms alongside cognitive functioning among Indian adults aged 45 years and older, utilizing nationally representative data and rigorously adjusting for a set of covariates accounting for potential confounding. Grounded in the context described earlier, we have formulated a conceptual framework, the key elements of which are illustrated in Fig. 1.

## Data and methods

### Participants and design

**Fig. 2:**
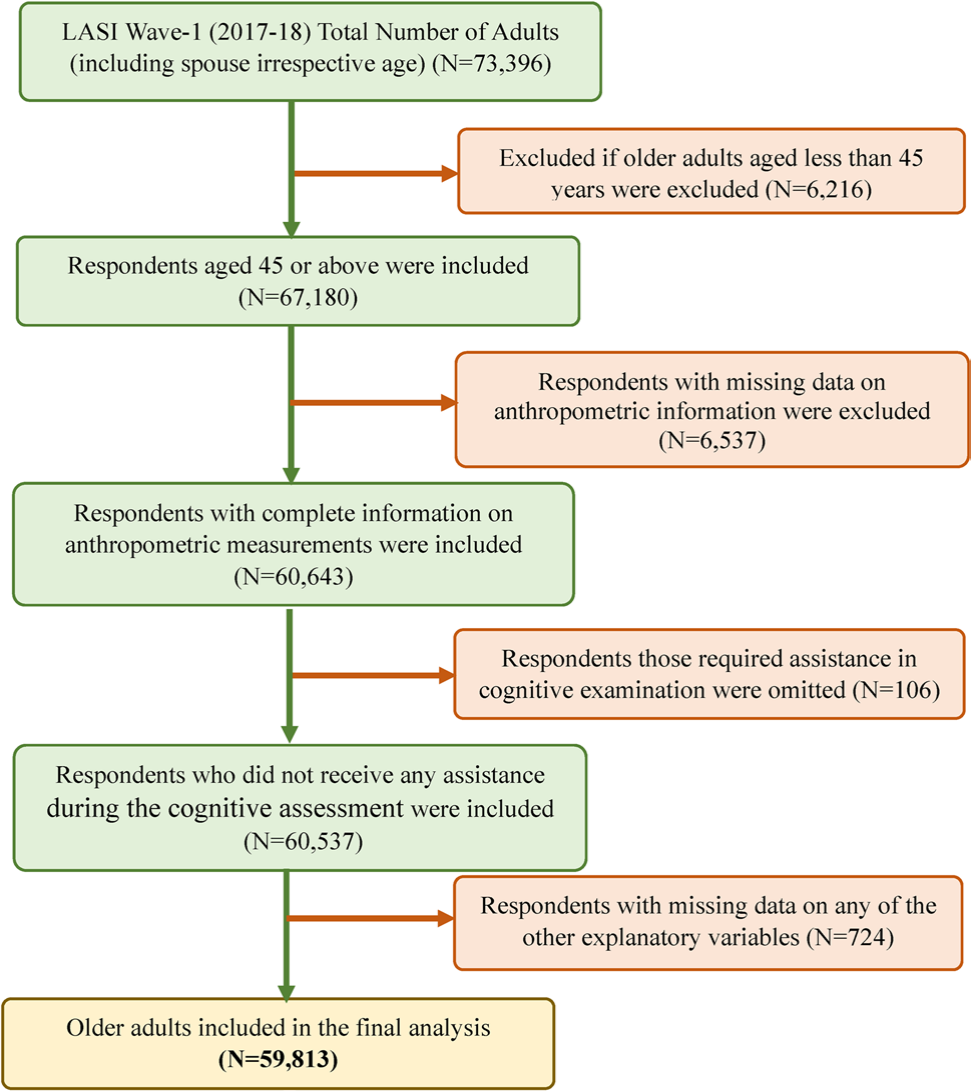
Selection of Study Sample Flow Diagram for the Present Study

Our study has utilized individual dataset of the Longitudinal Ageing Study in India (LASI 1st wave, 2017–2018), a nationally representative study undertaken by the International Institute for Population Sciences (IIPS) in Mumbai, under the administrative supervision of the Ministry of Health and Family Welfare, Government of India [33]. This survey employs a multistage, stratified cluster sampling approach, using a three-stages design for rural regions and a four-stage design in urban regions. The initial phase involved selecting sub-districts, known as Tehsils or Talukas. Subsequently, villages were chosen from rural areas within these sub-districts, while urban wards were selected from urban areas. In rural settings, the final stage entailed randomly selecting 32 households from each chosen village. In the case of the urban sampling process, an additional step was introduced: first, within every urban ward, each Census Enumeration Block (CEB) was chosen using random sampling, followed by the selection of 35 households from each CEB. This meticulous sampling strategy aimed to capture a diverse and representative snapshot of the ageing population in India across different geographical and socioeconomic strata [33]. Based on the objectives, individuals aged 45 years or older were deemed eligible for the study, resulting in 60,643 respondents after excluding those under 45 (*N* = 6,216). Respondents with missing anthropometric information (*N* = 6,537) were excluded. Respondents with missing data for any of the explanatory variables including caste, marital status, living arrangements, etc. (*N* = 830) were also dropped from the study. The final analytical sample comprised of 59,813 older adults, which consisted of 27,739 males and 32,074 females. Estimates were weighted to account for the complex survey design. Figure 2 illustrates a flowchart outlining the final process of sample selection for the analysis.

### Ethical approval statement

The Longitudinal Ageing Study in India (LASI) was carried out under the ethical oversight and approval of the Indian Council of Medical Research (ICMR) and the Central Ethics Committee on Human Research (CECHR). All study protocols adhered strictly to the ethical standards and principles set forth in the Declaration of Helsinki and other applicable national research guidelines.

### Consent to participate

Data collection was carried out through a field survey by trained survey agencies that adhered to established Human Subjects Protection protocols. The survey agencies ensured that all eligible respondents provided written or verbal informed consent before interviews and biomarker assessments were conducted. Participants were comprehensively informed about the study’s objectives and procedures to ensure their voluntary and informed participation.

### Methods

#### Measuring depressive symptoms

This study has used the ten-item version of the Center for Epidemiological Studies-Depression (CES-D) scale to assess the existence of depressive symptoms in older adults in India [34, 35]. This scale has widely been employed in studies of depression in later life and exhibits robust psychometric reliability and validity for use in older populations. The ten major items in the CES-D scale comprise three constructive symptoms (feeling satisfied, happy and hopeful) and seven negative symptoms (sense of feeling alone, feeling low energy, depression, trouble concentrating, everything is an effort, bothered by things, and fear of something). The response options for these items were: never or rarely (less than one day), sometimes (1 to 2 days), often (3 to 4 days), and all or most of the time (5 to 7 days) during the week before the interview. For negative symptoms, “never or rarely” (< 1 day) and “sometimes” (1 to 2 days) were assigned a score of 0, while “often” (3 to 4 days), and “most or all of the time” (5 to 7 days) were coded as 1. Reverse coding was applied to positive symptom items. The total depressive symptom score, derived from summing the scores of 10 questions, ranges between 0 and 10, with a score value of 4 or above demonstrating the presence of depressive symptoms of clinical significance, measured using the 10-item scale [36].

#### Operationalization of cognitive function

Cognitive performance was assessed using the health and retirement survey (HRS) adapted cognitive module embedded in the LASI survey, capturing five functional domains with a total composite score ranging from 0 to 43, where higher the score reflects better performance. The memory domain contributed the largest share of the score (0–20 points), assessed through two recall tasks administered at different time points during the interview. Orientation to both time and place was evaluated through eight structured questions (0–8 points). Arithmetic ability was tested across three tasks: a serial subtraction exercise, a basic computation problem, and a backward counting task together yielding a maximum of nine points. Executive functioning was measured through a paper-folding command task and a visuo-constructive pentagon-drawing exercise (0–4 points combined), and object naming assessed the ability to identify two common items (0–2 points). Individuals with scores lower than the 10th centile were coded as 1, indicating cognitive impairment (CoI), while those with scores at or above the 10th centile were coded as 0 ‘No Cognitive Impairment (NCoI). In our study, individuals who received any form of external assistance received while completing the assessment were omitted from the study sample. Various studies conducted in community settings across China [37], India [38], the USA [39], and England [40] have previously demonstrated the reliability and robust validity of these cognitive domains in assessing cognitive performance among older adults.

#### Assessment of physical activities

To measure vigorous activities the individuals were inquired about the type and frequency of activities in their daily life which include “*swimming*,* running or jogging*,* cycling*,* attending a health centre or gym*,* heavy lifting*,* digging with a spade or shovel*,* chopping*,* farm work*,* fast bicycling*,* and cycling with loads*” [33]. The answers were recorded as every day, more frequent than once a week, one time a week, one to three times monthly or seldom/never. Every day was coded as “Yes”, while all other categories were coded as “No.”

Moderate activities were measured using the question on engagement in moderately energetic activities included “*washing clothes by hand*,* drawing water from a well*,* cleaning house*,* fetching water or wood*,* gardening*,* bicycling at a regular pace*,,* floor or stretching exercises*,* walking at a moderate pace*,* and dancing*” were also asked respondents [33]. A response of every day engagement was considered as “Yes” otherwise “No”.

Holistic well-being activities were assessed by questioning respondents, “*How frequently do you participate in practices such as meditation*,* yoga*,* asana*,* pranayama*,* or related activities?”* [33]. The responses were recoded as “Yes” if the participant reported engaging in these activities every day, and “No” otherwise.

The selection of ‘*Every day*’ as the threshold for all three activities was intended to capture regular and sustained engagement in physical activity thereby distinguishing habitual behaviour from occasional participation.

#### Covariates

The analytical framework incorporated a comprehensive set of demographic and socioeconomic covariates to account for potential confounding. Sex was incorporated as a binary variable distinguishing male and female respondents. Age was grouped into three categories reflecting distinct life stages: middle-aged adults (45–59 years), older adults (60–74 years), and the oldest-old (75 years and above). Educational attainment was stratified into four levels based on years of formal schooling completed: none, fewer than five years, five to nine years, and ten or more years [41]. Based on the information of current marital status, respondents were categorized as ‘currently in union’ and the second category coded as ‘not in union’ which included widowed, divorced, separated, and never married. The Monthly Per Capita Consumption Expenditure (MPCE) was estimated through consumption data of household and categorized in five quintiles (Q1: Poorest, Q2: Poorer, Q3: Middle, Q4: Richer, and Q5: Richest) [33]. Body Mass Index (BMI) was categorized as underweight (BMI < 18.5 kg/m²), normal weight (18.5–24.9 kg/m²), overweight (25.0–29.9 kg/m²), and obese (≥ 30.0 kg/m²) according to the World Health Organization’s (WHO) classification [42]. The domicile place was recoded as urban and rural. The living arrangement of the participants was classified into five categories such as “living alone”, “living with spouse and children”, “living with spouse”, “living with children and others” and “living with others only” which includes other family members/relatives [33]. Work status was classified as, “currently working”, “retired” and “never worked”. The variable of social participation was constructed using responses to six items that assessed the frequency of engaging in various social activities. These included attending meetings or gatherings of organizations, clubs, or societies; visiting friends and relatives; attending cultural events such as cultural performances or cinema; engagement in religious events like bhajans, satsangs, or prayers; attending political, community, or organizational meetings; visiting relatives or friends; and meeting with friends. Each activity was recoded on a scale from 0 to 4, where 0 (never) to 4 (daily), with intermediate values indicating participation at least once a year (1), once a month (2), or once a week (3). The scores from all six items were combined to create a composite social participation score ranging from 0 to 24. This score was then categorized into three percentile-based groups coded as “low”, “moderate”, and “high” levels of social participation using the first, second, and third terciles.

### Analytical strategy

The descriptive statistical techniques were used to describe the study population, as well as estimate the weighted prevalence of vigorous, moderate, and holistic physical activities based on the selected sociodemographic characteristics. The analyses also focused on adjusted prevalence ratios (APRs) to examine the association between key outcomes and types of physical activities.

Multivariable Poisson regression models with robust standard errors were applied to derive adjusted prevalence ratios (APRs) for the major independent variables. This approach was preferred over odds ratios (OR) due to the high prevalence of some outcomes, where ORs tend to overestimate the association. The APRs were adjusted for potential covariates, including age, gender, MPCE quintile, education, and other relevant covariates.

$$\:{log}\left({\mu\:}_{i}\right)={\beta\:}_{0}+{\beta\:}_{1}{X}_{1i}+{\beta\:}_{2}{X}_{2i}+\dots\:+{\beta\:}_{k}{X}_{ki}$$

$\:{\mu\:}_{i}$ is the expected prevalence for individual i, $\:{X}_{1i},{X}_{2i}\dots\:,{X}_{ki}$ are covariates, and $\:{\beta\:}_{0},\:\:{\beta\:}_{1},\:\:{\beta\:}_{2},\dots\:{\beta\:}_{k}$ are the estimated coefficients. The adjusted prevalence ratio (PR) for each covariate is calculated as:

$$\:PR={e}^{{\upbeta\:}}$$

All the analyses factored in complex survey design by using corresponding sampling weights and a two-sided alpha level of 0.05 was applied to test statistical significance. All the data analysis for this study had been conducted using STATA version 17 [43] and R Studio version 4.5.1 [44].

## Results

### Patterns of physical activity by sociodemographic characteristics

Table 1 shows significant differences in engagement in PAs across various sociodemographic characteristics. Females were more likely to engage in moderate PA (62.3%) compared to males (37.7%), while males had higher engagement in vigorous PA (60.9% vs. 39.0%). Engagement in holistic well-being activities was slightly higher among males (51.3%) than females (48.7%). Participation in both moderate and vigorous physical activities decreased with age, with 56.9% of those aged 45–59 engaging in moderate activity compared to only 4.6% among those 75+, and 63.2% of the younger age group engaging in vigorous activity compared to just 3.1% of the oldest. Wealthier individuals showed lower engagement in physical activities, but were more involved in holistic well-being activities, with 24.3% of the richest quintile participating. Those with 10 or more years of schooling had greater engagement in holistic well-being activities (35.4%) compared to those with no education (29.9%). Rural residents were more likely to engage in moderate (69.2%) and vigorous physical activities (75%) than urban residents. Individuals in union (75.4% for moderate, 84% for vigorous) and those living with a spouse and others (73.6% for moderate, 82.3% for vigorous) were more likely to participate in physical activities.

**Table 1:** Weighted Proportion (%) of Moderate, Vigorous and Holistic Well-being Physical Activities across Various Background Characteristics of Study Population, India (*N* = 59,813)

Variables	Engaged in Moderate PA	*p*-value		Engaged in Vigorous PA	*p*-value	Holistic Well-being Activities*		*p*-value
	w% (95% CI)			w% (95% CI)		w% (95% CI)		
Gender		< 0.001			< 0.001			< 0.001
Male	37.7 (36.4–38.9)		60.9 (58.9–62.9)			51.3 (48.3–54.3)		
Female	62.3 (61.1–63.6)		39.0 (37.1–41)			48.7 (45.7–51.7)		
Age groups		< 0.001			< 0.001			< 0.001
45–59	56.9 (55.5–58.2)			63.2 (61.5–65)		48.2 (45.2–51.2)		
60–74	38.6 (37.3–39.9)			33.7 (32.1–35.3)		43.3 (40.6–46)		
75+	4.6 (4.2-5)			3.1 (2.3–4.1)		8.5 (7.4–9.8)		
Wealth Quintile		0.106			< 0.001		< 0.001	
Poorest	21 (20.2–21.9)		20 (18.7–21.3)			12.4 (11.1–13.9)		
Poorer	21.5 (20.6–22.3)		21.8 (20.7–23)			17.8 (16.2–19.6)		
Middle	20.2 (19.2–21.2)		20.3 (19-21.7)			22.6 (18.9–26.7)		
Richer	19.6 (18.4–20.8)		20.6 (18.8–22.5)			22.9 (21.2–24.8)		
Richest	17.7 (16.4–19.1)		17.3 (15.4–19.3)			24.3 (22.4–26.2)		
Years of education		< 0.001			< 0.001		< 0.001	
No education	50.6 (49.2–51.9)		46.9 (45.1–48.8)			29.9 (27.8–32.1)		
less than 5 years	11.2 (10.6–11.8)		12.6 (11.6–13.6)			10.6 (9.5–11.9)		
5–9 years	20.9 (19.8–21.9)		21.2 (20-22.5)			24.1 (22.2–26.1)		
10 or more years	17.4 (15.8–19)		19.3 (17-21.8)			35.4 (31.9–38.9)		
BMI category		< 0.001			< 0.001		< 0.001	
Underweight	20.4 (19.5–21.4)		21.4 (19.7–23.2)			13 (11.7–14.5)		
Normal	51.3 (49.9–52.6)		53.5 (51.5–55.6)			47.9 (45-50.8)		
Overweight	21.2 (19.8–22.6)		20.7 (18.7–23)			28.6 (25-32.5)		
Obese	7.1 (6.2–8.2)		4.3 (3.7–5.1)			10.5 (9.4–11.8)		
Place of residence		< 0.001			< 0.001		< 0.001	
Rural	69.2 (67.5–70.8)		75 (72.4–77.4)			59.4 (56.1–62.7)		
Urban	30.8 (29.2–32.5)		25 (22.6–27.6)			40.6 (37.3–43.9)		
Marital status		< 0.001			< 0.001		< 0.001	
In union	75.4 (74.1–76.8)		84 (82.1–85.7)			80.2 (78.5–81.8)		
Not in union	24.6 (23.2–25.9)		16 (14.3–17.9)			19.8 (18.2–21.5)		
Living arrangements		< 0.001			< 0.001		< 0.001	
Living alone	4.4 (4-4.8)			2.7 (2.2–3.3)		1.9 (1.5–2.4)		
Living with spouse	16.5 (15.8–17.3)			15.7 (14.7–16.8)		14.1 (12.8–15.6)		
Living with spouse and children	56.9 (55.5–58.2)			66.5 (64.6–68.3)		64.8 (62.4–67.2)		
Living with children and others	18.1 (16.9–19.4)			11.2 (10.3–12.2)		16.0 (14.6–17.5)		
Living with others only	4.1 (3.3–4.9)			3.8 (2.4-6.0)		3.1 (2.4–3.8)		
Work status		< 0.001			< 0.001		< 0.001	
Never worked	27.5 (26.1–28.9)		10.9 (9.0–13.0)			31.4 (27.8–35.1)		
Currently working	51.5 (50.2–52.8)		78.6 (76.5–80.4)			39.3 (36.8–41.9)		
Retired	20.9 (20.2–21.8)		10.5 (9.7–11.5)			29.3 (27.2–31.6)		
Social Participation		< 0.001			< 0.001			< 0.001
Low	33.3 (32.2–34.4)			29.3 (27.9–30.8)		23.4 (21.5–25.4)		
Moderate	42.5 (41.2–43.8)			40.7 (38.8–42.6)		44.4 (41.2–47.6)		
High	24.2 (23.1–25.2)			29.9 (28.1–31.9)		32.2 (29.9–34.6)		

*Holistic wellbeing activities - yoga, meditation, asana, pranayama etc

*PA* Physical activity, *w%* weighted prevalence, *CI* Confidence interval

Individuals co-residing with their spouse and children reported higher engagement in both moderate (56.9%) and vigorous (66.5%) physical activities, as well as holistic well-being activities (64.8%). In contrast, those living alone had lower participation across all activity types, especially in holistic well-being activities (1.9%). Social participation was positively associated with all three types of activities. Individuals with high levels of social participation showed greater engagement in moderate (24.2%) and vigorous (29.9%) physical activity and notably higher participation in holistic well-being activities (32.2%) compared to those with low social participation (23.4%). Regarding work status, currently working individuals had higher participation in both moderate (51.5%) and vigorous (78.6%) physical activities compared to their counterparts, those who never worked.

### Prevalence of mental health outcomes by physical activity status among Indian adults

**Fig. 3:**
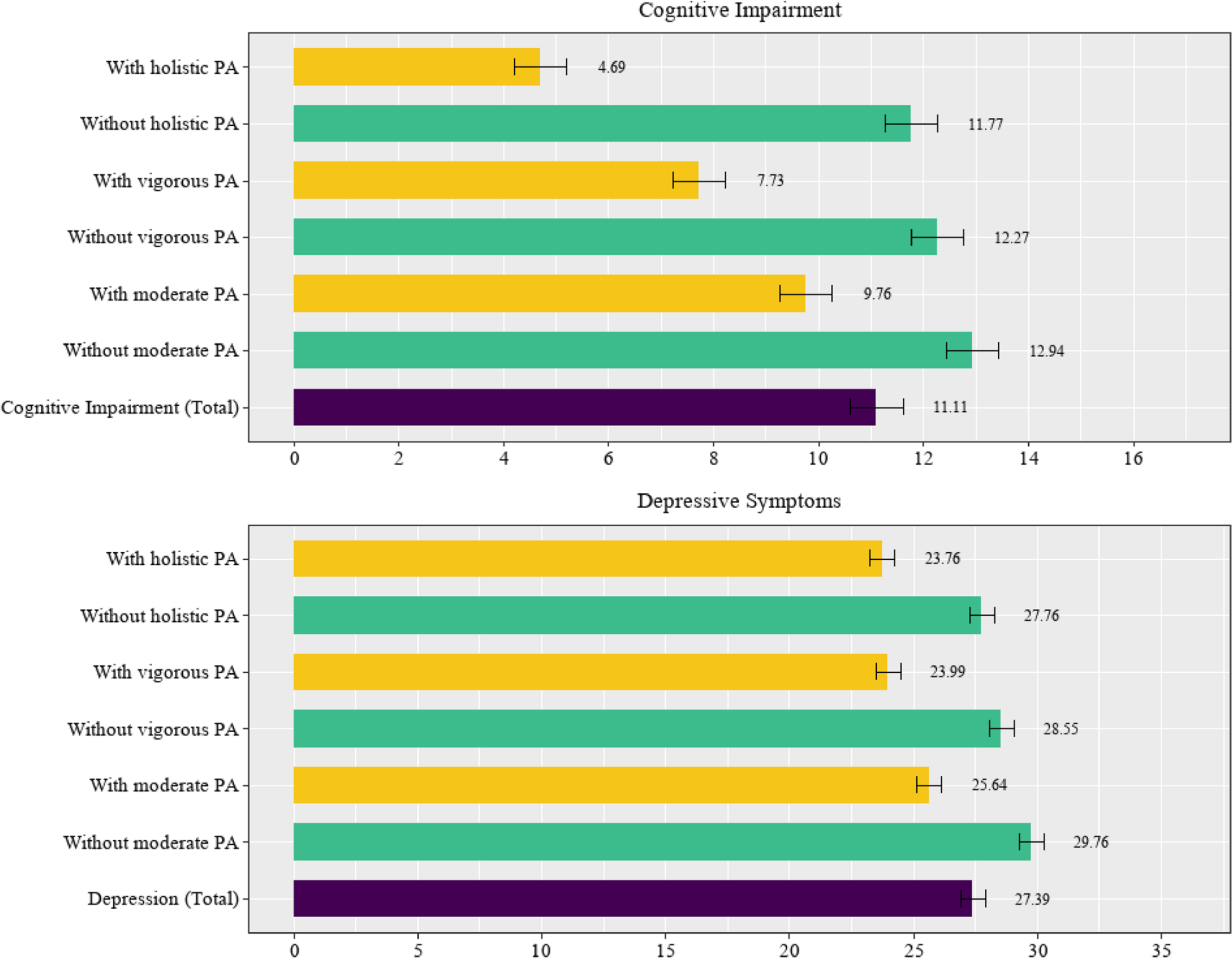
Prevalence of Cognitive Impairment and Depressive Symptoms by Engagement in Holistic, Moderate and Vigorous Physical Activities among Older Adults in India. Note: PA: Physical activity; Data Source: Longitudinal Ageing Study in India (Wave-1), 2017-18

Figure 3 reveals the prevalence of CoI and depressive symptoms among Indian older adults, categorized by their engagement in various physical activities. For CoI, individuals without holistic PA show a higher prevalence (11.77%) compared to those with holistic PA (4.69%). Likewise, those without vigorous PA had a higher prevalence (12.27%) than those with vigorous PA (7.72%). The difference was also notable for moderate PA, with 12.94% prevalence for those without and 9.76% for those with moderate PA. The overall prevalence of CoI is 11.11%. Regarding depressive symptoms, the pattern was similar but with higher percentages overall. Those without holistic PA showed 27.76% prevalence versus 23.76% for those with holistic PA. For vigorous PA, the prevalence was 28.55% without and 23.99% with. Moderate PA showed 29.76% prevalence without and 25.64% with. The overall prevalence of depressive symptoms was reported 27.39%.

### Multivariable analysis of factors associated with cognitive impairment among Indian adults

Table 2 indicates that 58.4% of individuals without CoI engage in moderate physical activity, compared to 50.5% of those with CoI. For vigorous PA, 26.5% of individuals without CoI participate, while only 17.7% of those with CoI do. Engagement in holistic well-being activities was low for both groups, but notably lower among those with CoI (3.9%) compared to those without (10%). The association between CoI and these activities indicates that individuals not engaging in moderate PA had a higher likelihood of depressive symptoms, with an adjusted PR of 1.17 (95%CI: 1.08,1.26). Similarly, those not engaged in vigorous PA initially showed a significant association with CoI (APR:1.63, 95%CI: 1.43,1.86), but this association weakens after adjustment (APR:1.07, 95%CI: 0.94,1.22). Engagement in holistic well-being activities was strongly associated with a lower likelihood of CoI, with an adjusted PR of 1.42 (95%CI: 1.22,1.67). Living arrangements showed variation in cognitive outcomes. Although the proportion of older adults with CoI was lower among individuals cohabiting with their spouse and children (34.3%), individuals living alone, despite forming a smaller subgroup, had a relatively higher rate of CoI within that group (6.6%). Importantly, those living with others only showed a significantly higher likelihood of CoI (APR: 1.21, 95% CI: 1.03–1.46) compared to those living alone. Individuals who were currently working had a lower prevalence (30.5%) and decreased likelihood of CoI (APR: 0.89, 95% CI: 0.77–0.96). In contrast, those who were retired had a higher prevalence (38.1%) and a greater likelihood of impairment (APR: 1.10, 95% CI: 1.01–1.21). High social participation was associated with a significantly lower prevalence of CoI (9.6%) and an APR of 0.61 (95% CI: 0.53–0.70).

**Table 2:** Weighted Prevalence (%) of Cognitive Impairment According to Different Types of Physical Activity and Selected Sociodemographic Characteristics, India (*N* = 59,813)

Measures	Cognitive Impairment			Model 1	Model 2	Model 3	Model 4
	No	Yes	*p* ^a^	UPR (95% CI) ^b^	APR (95% CI) ^c^	APR (95% CI) ^c^	APR (95% CI) ^c^
Engaged in Moderate PA			< 0.001				
Yes	58.4 (57.5–59.2)	50.5 (48.4–52.6)		1	1		
No	41.6 (40.8–42.5)	49.5 (47.4–51.6)		1.38*** [1.27,1.50]	1.12*** [1.04,1.21]		
Engaged in Vigorous PA			< 0.001				
Yes	26.5 (25.6–27.4)	17.7 (15.9–19.8)		1		1	
No	73.5 (72.6–74.4)	82.3 (80.2–84.1)		1.63*** [1.43,1.86]		1.09** [1.04,1.12]	
Engaged in Holistic Well-being Activity			*< 0.001*				
Yes	10 (9.5–10.6)	3.9 (3.4–4.6)		1			1
No	90 (89.4–90.5)	96.1 (95.4–96.6)		2.56*** [2.16,3.04]			1.36*** [1.16,1.59]
Gender			< 0.001				
Male	48.6 (47.6–49.5)	23.6 (21.9–25.3)		0.38*** [0.35,0.42]	0.61*** [0.56,0.68]	0.63*** [0.57,0.69]	0.63*** [0.58,0.70]
Female	51.4 (50.5–52.4)	76.4 (74.7–78.1)		1	1	1	1
Age Groups			< 0.001				
45–59	52.9 (52-53.9)	27.2 (25.4–29)		1	1	1	1
60–74	40.4 (39.5–41.4)	50.4 (48.3–52.5)		2.21*** [2.00,2.45]	1.49*** [1.36,1.63]	1.51*** [1.37,1.65]	1.51*** [1.37,1.66]
75+	6.6 (6.2–7.1)	22.4 (20.7–24.2)		4.92*** [4.39,5.51]	2.36*** [2.07,2.68]	2.42*** [2.14,2.75]	2.43*** [2.14,2.75]
Wealth Quintile			< 0.001				
Poorest	20.2 (19.6–20.9)	27.9 (26.2–29.7)		2.00*** [1.73,2.32]	1.20* [1.05,1.38]	1.19* [1.04,1.37]	1.18* [1.03,1.36]
Poorer	20.9 (20.2–21.5)	24.5 (22.8–26.4)		1.71*** [1.47,2.00]	1.18* [1.02,1.35]	1.17* [1.01,1.35]	1.16* [1.01,1.33]
Middle	20.5 (19.7–21.2)	19.5 (17.9–21.2)		1.50*** [1.27,1.77]	1.06 [0.92,1.23]	1.06 [0.91,1.23]	1.06 [0.91,1.23]
Richer	20 (19.2–20.9)	16.6 (14.9–18.4)		1.27** [1.06,1.52]	1.03 [0.89,1.20]	1.04 [0.88,1.22]	1.03 [0.88,1.20]
Richest	18.4 (17.4–19.3)	11.4 (10.1–13)		1	1	1	1
Years of Education			< 0.001				
No education	45.7 (44.8–46.6)	90.6 (89.4–91.7)		47.21*** [30.91,72.11]	21.62*** [13.60,34.34]	21.75*** [13.68,34.57]	21.14*** [13.29,33.62]
less than 5 years	11.8 (11.3–12.2)	6.4 (5.5–7.5)		15.24*** [9.74,23.85]	9.49*** [5.87,15.35]	9.50*** [5.87,15.38]	9.30*** [5.74,15.07]
5–9 years	23 (22.2–23.8)	2.4 (2–3)		3.46*** [2.18,5.49]	2.45*** [1.49,4.04]	2.45*** [1.49,4.04]	2.41*** [1.46,3.97]
10 or more years	19.6 (18.5–20.7)	0.6 (0.4-1)		1	1	1	1
BMI Category			< 0.001				
Underweight	19.3 (18.6–20)	38.5 (36.5–40.5)		1	1	1	1
Normal	51.8 (50.8–52.8)	49.2 (47.1–51.3)		0.53*** [0.49,0.58]	0.78*** [0.72,0.84]	0.78*** [0.72,0.84]	0.78*** [0.72,0.84]
Overweight	21.6 (20.7–22.6)	9.5 (8.1–11)		0.26*** [0.22,0.31]	0.55*** [0.47,0.65]	0.55*** [0.47,0.65]	0.55*** [0.47,0.65]
Obese	7.3 (6.7-8)	2.9 (2-4.2)		0.24*** [0.16,0.35]	0.45*** [0.35,0.57]	0.45*** [0.36,0.57]	0.45*** [0.36,0.58]
Place of Residence			< 0.001				
Rural	67.9 (66.8–69)	85.6 (83.5–87.4)		1	1	1	1
Urban	32.1 (31-33.2)	14.4 (12.6–16.5)		0.42*** [0.36,0.49]	0.71*** [0.62,0.82]	0.71*** [0.62,0.82]	0.73*** [0.63,0.84]
Marital Status			< 0.001				
In union	77.2 (76.3–78.1)	51.5 (49.4–53.6)		1	1	1	1
Not in union	22.8 (21.9–23.7)	48.5 (46.4–50.6)		2.69*** [2.47,2.92]	1.56*** [1.14,2.14]	1.59*** [1.16,2.17]	1.58** [1.16,2.16]
Living Arrangements			< 0.001				
Living alone	3.3 (3.1–3.6)	6.6 (5.8–7.6)		1	1	1	1
Living with spouse	15.9 (15.4–16.6)	15.8 (14.3–17.5)		0.54*** [0.46–0.64]	0.46*** [0.23,0.89]	1.49* [1.06,2.11]	1.49* [1.06,2.10]
Living with spouse and children	59.6 (58.7–60.5)	34.3 (32.6–36.3)		0.34*** [0.29–0.39]	1.19 [0.85,1.66]	1.23 [0.88,1.72]	1.23 [0.88,1.72]
Living with children and others	17.3 (16.5–18.2)	35.5 (33.5–37.5)		1.03 [0.89–1.19]	1.09 [0.95,1.25]	1.11 [0.97,1.27]	1.12 [0.97,1.23]
Living with others only	3.7 (3.2–4.3)	7.7 (6.6–8.9)		1.05 [0.85–1.29]	1.19*** [1.12,1.33]	1.21* [1.02,1.43]	1.21** [1.03,1.46]
Work Status			< 0.001				
Never worked	25.6 (24.6–26.7)	31.3 (29.5–33.1)		1	1	1	1
Currently working	49.1 (48.2–50.0)	30.5 (28.5–32.6)		0.54*** [0.48–0.60]	0.88** [0.79,0.98]	0.86** [0.78,0.96]	0.89** [0.77,0.96]
Retired	25.3 (24.8–25.9)	38.1 (36.1–40.1)		1.20*** [1.09–1.33]	1.11* [1.02,1.22]	1.11* [1.02,1.21]	1.10* [1.01,1.21]
Social Participation			< 0.001				
Low	34.9 (34.1–35.7)	54.8 (52.7–56.9)		1	1	1	1
Moderate	41.3 (40.3–42.3)	35.5 (33.5–37.6)		0.59*** [0.54–0.65]	0.86*** [0.80,0.94]	0.86*** [0.79,0.93]	0.86*** [0.80,0.94]
High	23.8 (23.0-24.6)	9.6 (8.3–11.1)		0.29*** [0.25–0.34]	0.62*** [0.54,0.71]	0.61*** [0.53,0.70]	1.61*** [0.53,0.71]

ᵃ p-values are based on chi-square tests; ᵇ UPR: unadjusted prevalence ratios estimated using Poisson regression; ᶜ APR: adjusted prevalence ratios estimated using Poisson regression with robust error variance and backward stepwise selection. Model 1 presents unadjusted estimates; Models 2–4 include moderate, vigorous, and holistic physical activity, respectively, adjusted for demographic, socioeconomic, and health-related covariates

*CI* Confidence interval, *PA* Physical activity

Reference category is indicated as “1”. *p < 0.05, **p < 0.01, ***p < 0.001

### Multivariable analysis of factors associated with depressive symptoms among Indian adults

Similarly, Table 3 reveals that 58.9% of individuals without depressive symptoms engage in moderate PA, compared to 53.8% of those with depressive symptoms. Further, 26.7% of individuals without depressive symptoms participate in vigorous PA, while only 22.4% of those with depressive symptoms do. For holistic well-being activities, 9.8% of individuals without depressive symptoms were engaged, compared to 8.1% of those with depressive symptoms. The correlation between depressive symptoms and PA showed that individuals not engaged in moderate PA were significantly more likely to experience depressive symptoms, with an unadjusted PR of 1.18 (95%CI: 1.12,1.24). A similar trend was observed for vigorous PA, where those not engaged have an unadjusted PR of 1.20 (95%CI: 1.12,1.28), indicating a higher likelihood of depressive symptoms. However, there was no significant association between holistic well-being activities and depressive symptoms after adjustment (adjusted PR: 1.05), suggesting that engagement in these activities was not significantly correlated with depressive symptoms after adjustment. Marital status was a significant factor, with those not in a union showing 24% higher prevalence (APR: 1.24, 95% CI: 1.05–1.46), compared to those were in a union. Those living alone had the highest prevalence of depressive symptoms. In contrast, individuals living with a spouse and children (APR: 0.77, 95% CI: 0.64–0.91), with children and others (APR: 0.73, 95% CI: 0.66–0.80), or with others only (APR: 0.81, 95% CI: 0.70–0.94) had a significantly lower likelihood of depressive symptoms compared to individuals who were living alone. Participants who were currently working had a lower likelihood (APR: 0.91, 95% CI: 0.85–0.99), while those who were retired had a higher likelihood (APR: 1.10, 95% CI: 1.03–1.19) of experiencing depressive symptoms, relative to those who never worked. Older adults with moderate and high social participation had slightly lower prevalence of depressive symptoms in comparison with individuals with low social engagement.

**Table 3:** Weighted Prevalence (%) of Depressive Symptoms According to Different Types of Physical Activity and Selected Sociodemographic Characteristics, India (*N* = 59,813)

Measures	Depressive Symptoms			Model 1	Model 2	Model 3	Model 4
	No	Yes	*p* ^a^	UPR (95% CI) ^b^	APR (95% CI) ^c^	APR (95% CI) ^c^	APR (95% CI) ^c^
Engaged in Moderate PA			< 0.001				
Yes	58.9 (57.9–59.8)	53.8 (52.3–55.3)		1	1		
No	41.1 (40.2–42.1)	46.2 (44.7–47.7)		1.18*** [1.11,1.24]	1.17*** [1.11,1.23]		
Engaged in Vigorous PA			< 0.001				
Yes	26.7 (25.7–27.8)	22.4 (21.1–23.6)		1		1	
No	73.3 (72.2–74.3)	77.6 (76.4–78.9)		1.20*** [1.12,1.28]		1.09*** [1.05,1.12]	
Engaged in Holistic Well-being Activity			< 0.001				
Yes	9.8 (9.4–10.3)	8.1 (6.7–9.8)		1			1
No	90.2 (89.7–90.6)	91.9 (90.2–93.3)		1.18 [1.00,1.39]			1.06 [0.90,1.24]
Gender			< 0.001				
Male	47.6 (46.6–48.7)	40.9 (39.4–42.4)		0.82*** [0.78,0.87]	0.90*** [0.85,0.96]	0.94* [0.87,1.00]	0.94** [0.87,0.98]
Female	52.4 (51.3–53.4)	59.1 (57.6–60.6)		1	1	1	1
Age Groups			< 0.001				
45–59	51.4 (50.3–52.5)	46.5 (44.9–48.2)		1	1	1	1
60–74	40.8 (39.7–41.8)	43.6 (42.1–45.2)		1.13*** [1.06,1.20]	1.01 [0.96,1.07]	1.00 [0.96,1.01]	1.04 [0.99,1.12]
75+	7.8 (7.3–8.4)	9.8 (9.1–10.7)		1.29*** [1.18,1.41]	1.03 [0.93,1.12]	1.07 [0.95,1.11]	1.08 [0.98,1.17]
Wealth Quintile			< 0.001				
Poorest	20.4 (19.7–21.2)	22.9 (21.7–24.1)		1.14** [1.04,1.24]	1.05 [0.96,1.15]	1.04 [0.96,1.14]	1.04 [0.95,1.14]
Poorer	21.3 (20.6–22)	21.2 (20.1–22.3)		1.04 [0.95,1.14]	0.98 [0.90,1.07]	0.98 [0.90,1.07]	0.98 [0.90,1.07]
Middle	20.2 (19.5–20.9)	21 (19.3–22.7)		1.08 [0.97,1.20]	1.03 [0.92,1.15]	1.02 [0.91,1.14]	1.02 [0.91,1.15]
Richer	20.2 (19.2–21.2)	18.2 (17.2–19.3)		0.97 [0.88,1.07]	1.00 [0.85,1.02]	0.94 [0.86,1.03]	0.94 [0.85,1.03]
Richest	17.9 (16.8–19)	16.8 (15.7–18)		1	1	1	1
Years of Education			< 0.001				
No education	48.1 (47-49.1)	57.6 (56-59.3)		1.54*** [1.34,1.77]	1.32*** [1.14,1.52]	1.33*** [1.15,1.53]	1.32*** [1.15,1.51]
less than 5 years	11.2 (10.7–11.7)	11.1 (10.3–12)		1.35*** [1.16,1.57]	1.23** [1.06,1.42]	1.23** [1.07,1.43]	1.22** [1.06,1.41]
5–9 years	21.6 (20.7–22.5)	18.2 (17.2–19.3)		1.20* [1.03,1.38]	1.13 [0.98,1.32]	1.13 [0.98,1.31]	1.13 [0.98,1.31]
10 or more years	19.2 (18-20.4)	13 (11.3–14.9)		1	1	1	1
BMI Category			< 0.001				
Underweight	20 (19.1–20.8)	25.2 (24-26.4)		1	1	1	1
Normal	51.9 (50.8–53)	50.4 (48.8–52)		0.83*** [0.79,0.88]	0.89*** [0.84,0.94]	0.89*** [0.84,0.94]	0.89*** [0.85,0.95]
Overweight	20.8 (19.8–21.9)	18.8 (17.1–20.7)		0.79*** [0.71,0.87]	0.88** [0.80,0.97]	0.88** [0.81,0.97]	0.89** [0.81,0.97]
Obese	7.3 (6.5–8.1)	5.5 (4.8–6.3)		0.69*** [0.60,0.80]	0.77*** [0.66,0.89]	0.77*** [0.66,0.89]	0.77** [0.67,0.90]
Place of Residence			< 0.001				
Rural	68.8 (67.6–70.1)	72.7 (70.8–74.5)		1	1	1	1
Urban	31.2 (29.9–32.4)	27.3 (25.5–29.2)		0.87 [0.81,0.95]	0.98 [0.91,1.05]	0.97 [0.90,1.04]	0.97 [0.91,1.04]
Marital Status			< 0.001				
In union	76.8 (75.7–77.9)	67.8 (66.4–69.2)		1	1	1	1
Not in union	23.2 (22.1–24.3)	32.2 (30.8–33.6)		1.37*** [1.29,1.45]	1.23* [1.04,1.45]	1.26** [1.07,1.48]	1.26** [1.07,1.49]
Living Arrangements			< 0.001				
Living alone	12.9 (2.5–3.2)	15.9 (5.4–6.5)		1	1	1	1
Living with spouse	15.8 (15.2–16.5)	16.2 (15.2–16.5)		0.62*** [0.56,0.69]	0.83*** [0.69,0.89]	0.85** [0.71,0.92]	0.86** [0.65,0.93]
Living with spouse and children	18.2 (17.3–19.1)	22.4 (21.1–23.7)		0.55*** [0.50,0.60]	0.73*** [0.61,0.88]	0.76** [0.64,0.91]	0.77** [0.64,0.91]
Living with children and others	59.3 (58.2–60.3)	50.2 (48.6–60.4)		0.72*** [0.65,0.79]	0.71*** [0.64,0.78]	0.72*** [0.66,0.80]	0.73***[0.66,0.80]
Living with others only	3.8 (3.2–4.5)	5.1 (4.6–5.8)		0.77** [0.65,0.90]	0.79*** [0.69,0.92]	0.81** [0.70,0.93]	0.81** [0.70,0.94]
Work Status			< 0.001				
Never worked	25.9 (24.8–27.1)	27.0 (25.4–28.7)		1	1	1	1
Currently working	48.8 (47.8–49.8)	42.3 (40.7–43.8)		0.87*** [0.80,0.94]	0.94* [0.86,0.98]	0.94 [0.86,1.02]	0.91* [0.85,0.99]
Retired	25.2 (24.4–25.9)	30.6 (29.3–32.0)		1.10* [1.02,1.20]	1.11** [1.03,1.19]	1.10** [1.03,1.19]	1.10** [1.03,1.19]
Social Participation			< 0.001				
Low	36.4 (35.5–37.3)	38.9 (37.4–40.3)		1	1	1	1
Moderate	40.8 (39.8–41.9)	28.2 (22.6–31.9)		0.94** [0.89,0.98]	0.97 [0.91,1.01]	1.02 [0.96,1.08]	1.02 [0.96,1.08]
High	22.7 (21.8–23.6)	20.8 (19.68-22.0)		0.89** [0.84,0.95]	1.03 [0.98,1.05]	1.04 [0.98,1.11]	1.04 [0.97,1.11]

ᵃ p-values are based on chi-square tests; ᵇ UPR: unadjusted prevalence ratios estimated using Poisson regression; ᶜ APR: adjusted prevalence ratios estimated using Poisson regression with robust error variance and backward stepwise selection. Model 1 presents unadjusted estimates; Models 2–4 include moderate, vigorous, and holistic physical activity, respectively, adjusted for demographic, socioeconomic, and health-related covariates

*CI* Confidence interval, *PA* Physical activity

Reference category is indicated as “1”. *p < 0.05, **p < 0.01, ***p < 0.001

### Differential impact of physical activities on depressive symptoms and cognitive impairment across age cohorts

Table 4 presents the age-stratified prevalence of depressive symptoms and cognitive impairment based on different physical activity profiles. For depressive symptoms (Panel A), the prevalence among individuals engaging in “0 Activities” rises dramatically from 42.2% in the middle-aged (45–59 years) group to 66.9% in the oldest-old (75 years and above). Conversely, the association between engaging in PA and lower risk of depression appeared strongest in the oldest cohort. For the 75 + group, participating in just one type of activity was associated with a sharp drop in depressive symptoms prevalence to 27.5% than younger age groups (29.4% and 38.7%). An analogous age trend had been be observed in case of CoI (Panel B). The prevalence for those with “0 Activities” climbs from 42.2% in the 45–59 age group to 67.2% in the 75 + group. Holistic activities demonstrated a strong and consistent protective association against CoI across all age groups, with prevalence rates remaining low and stable at 3.7%, 3.9%, and 4.2% for the youngest to oldest cohorts, respectively.

**Table 4:** Age-stratified Weighted Prevalence of Depressive Symptoms and Cognitive Impairment by Physical Activity Engagement Profiles among Indian Adults

Panel A: Depressive Symptoms (*N* = 59,813)			
Physical Activity Profiles	Middle-aged (45–59) (*N* = 31,465)	Older adults (60–74) (*N* = 23,708)	Oldest-old (75+) (*N* = 4,640)
	Weighted Prev. % (95% CI)	Weighted Prev. % (95% CI)	Weighted Prev. % (95% CI)
Individual Activities			
Moderate PA			
Yes	63.4 (61.2–65.6)	49.8 (47.7–51.9)	25.8 (22.4–29.3)
No	36.6 (34.4–38.7)	50.2 (48.0-52.3)	74.2 (70.6–77.5)
Vigorous PA			
Yes	28.5 (26.5–30.5)	19.6 (17.7–21.8)	5.5 (4.2–7.1)
No	71.5 (69.5–73.4)	80.3 (78.2–82.3)	94.5 (92.9–95.8)
Holistic Well-being Activity			
Yes	9.2 (6.5–13.1)	7.3 (6.5–8.2)	6.3 (4.8–8.1)
No	90.7 (86.9–93.4)	92.7 (91.8–93.4)	93.7 (91.9–95.2)
Cumulative Activities			
0 Activities	42.2 (39.6–44.7)	42.9 (40.9–45.1)	66.9 (64.1–71.5)
1 Type of Activity	29.4 (27.5–31.3)	38.7 (36.7–40.8)	27.5 (23.7–30.8)
2 Types of Activity	26.3 (23.4–29.5)	16.8 (14.8–18.9)	4.3 (3.3–5.8)
3 Types of Activity	2.1 (1.8–2.6)	1.5 (1.2–1.9)	1.3 (0.7–1.9)

Panel B: Cognitive Impairment (*N* = 59,813)			
Physical Activity Profiles	Middle-aged (45–59) (*N* = 31,465)	Older adults (60–74) (*N* = 23,708)	Oldest-old (75+) (*N* = 4,640)
	Weighted Prev. % (95% CI)	Weighted Prev. % (95% CI)	Weighted Prev. % (95% CI)
Individual Activities			
Moderate PA			
Yes	68.0 (64.5–71.2)	50.2 (47.1–53.2)	29.9 (25.6–34.6)
No	31.9 (28.8–35.3)	49.8 (46.7–52.8)	70.1 (65.3–74.4)
Vigorous PA			
Yes	33.5 (29.3–37.8)	14.7 (12.3–17.4)	11.1 (8.2–14.8)
No	66.5 (62.2–70.6)	85.3 (82.5–87.7)	88.9 (85.1–91.7)
Holistic Well-being Activity			
Yes	3.7 (2.7–5.1)	3.9 (3.1–4.9)	4.2 (2.9–5.8)
No	96.3 (94.9–97.3)	96.1 (95.0-96.9)	95.8 (94.1–97.0)
Cumulative Activities			
0 Activities	42.2 (39.5–46.9)	44.2 (42.2–48.4)	67.2 (62.6–71.5)
1 Type of Activity	26.2 (23.3–29.3)	41.4 (38.4–44.3)	25.7 (22.5–30.6)
2 Types of Activity	29.7 (25.6–34.1)	12.6 (10.3–15.2)	5.9 (3.4–10.0)
3 Types of Activity	1.9 (1.1–2.1)	1.8 (1.3-2.0)	1.2 (0.6–1.7)

*PA* Physical activity, *Holistic wellbeing activities - yoga, meditation, asana, pranayama etc., N - Sample size, *CI* Confidence interval

**Fig. 4:**
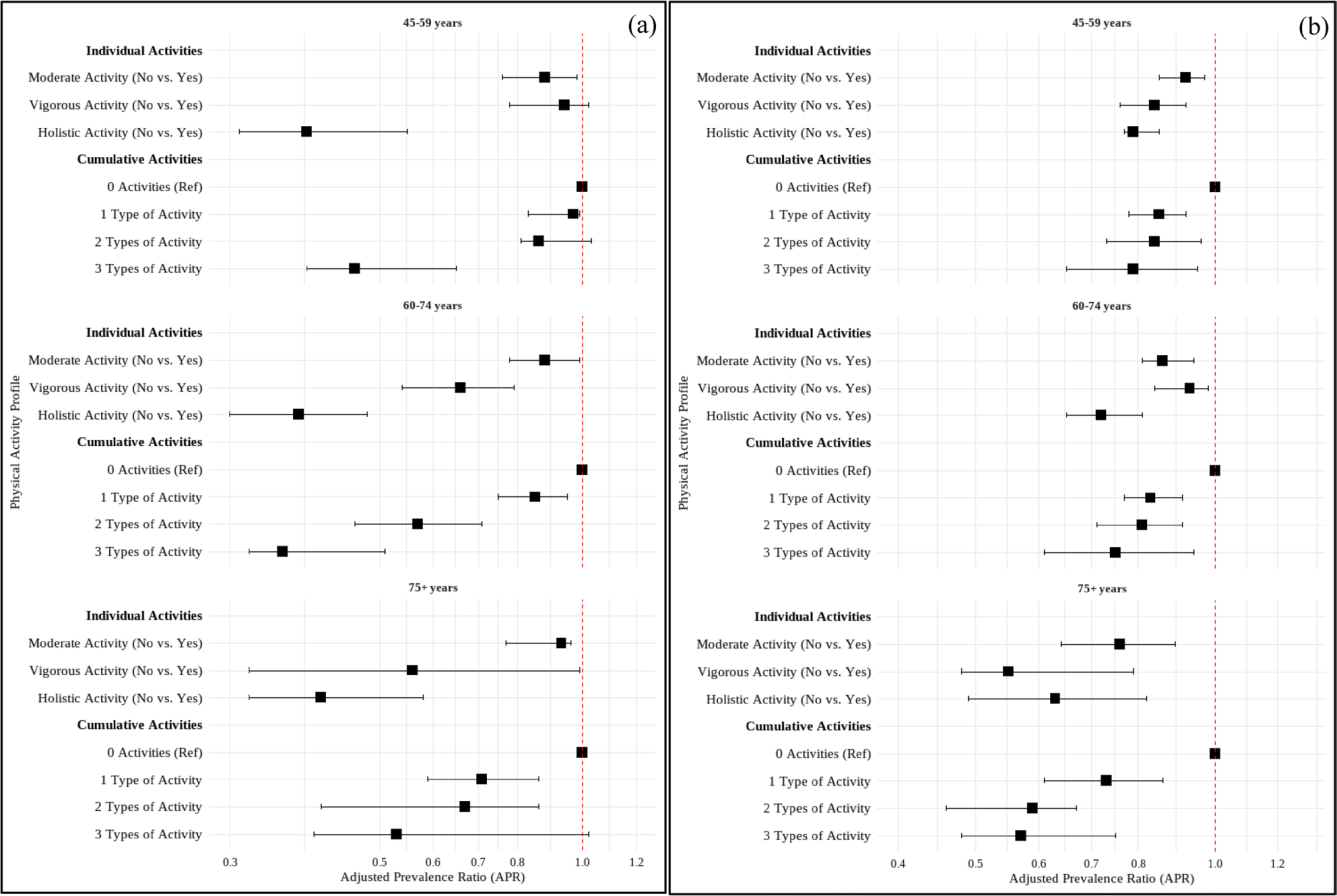
Age-stratified Adjusted Prevalence Ratios (APRs) for (**a**) Cognitive Impairment and (**b**) Depressive Symptoms by Physical Activity Profiles among Adults in India. Note: APR: Adjusted Prevalence Ratio, CI: Confidence Interval. All models are adjusted for relevant socio-demographic and health covariates. Data: Longitudinal Ageing Study in India (Wave-1), 2017-18

Figure 4 shows the APRs for depressive symptoms after controlling for covariates. Among the middle-aged (45–59 years), engaging in vigorous (APR: 0.84, 95% CI: 0.76–0.92) and holistic activities (APR: 0.79, 95% CI: 0.77–0.85) was significantly associated with a lower prevalence of depressive symptoms. In the 60–74 age group, holistic activity showed a strong protective association (APR: 0.72, 95% CI: 0.65–0.81), while the effect of vigorous activity was not statistically significant. Notably, the strongest protective associations were observed in the oldest-old (75 + years). In this group, vigorous (APR: 0.55, 95% CI: 0.48–0.79) and holistic activities (APR: 0.63, 95% CI: 0.49–0.82) were linked to a substantially lower prevalence of depressive symptoms. The cumulative analysis further reinforced this trend, showing that engaging in two (APR: 0.59, 95% CI: 0.46–0.67) or three types of activity (APR: 0.57, 95% CI: 0.48–0.75) was associated with the lowest prevalence of depressive symptoms among the oldest-old.

## Discussion

Our study identifies significant associations between PA and the mental health of older persons in India. The prevalence of depressive symptoms and cognitive decline was consistently lower among those who participated in PAs compared to those who did not perform PA regularly in India. Notably, the overall incidence of cognitive impairment was found to be around 11%, while depressive symptoms affected over a quarter of the studied population. Numerous studies have systematically established the positive association between regular PAs and the reduction of psychological and mood-related symptoms among adult and older populations [45–47]. To the best of our understanding, no prior epidemiological cross-sectional study in India has jointly examined both depressive symptoms and cognitive impairment in relation to PA frequency and key sociodemographic and anthropometric correlates, including gender, age, socioeconomic status, education, and BMI category.

The findings of this research align with prior investigations. Prior studies indicate that engaging in physical exercise is associated with a lower risk of cognitive deterioration [48, 49]. A comprehensive review of 18 controlled studies revealed that dementia patients who participated in more physical activities, particularly aerobic exercises, demonstrated enhanced cognitive functioning among older adults [50]. Several prospective cohort studies have indicated that older individuals who participate in vigorous PA tend to experience a slower cognitive decline over both long-term (up to 6 years) and short-term (around 2.5 years) [51] and [52] follow-up periods compared to those with lower levels of PA. According to Bherer, Erickson, and Liu-Ambrose (2013), exercise promotes structural changes in the brain, such as the formation of new blood vessels, neurons, and synapses. These changes contribute to improved functional capacity and cognitive abilities, including better executive functioning, faster information processing, and enhanced executive functioning in the older persons [49]. Tan et al. (2017) found a link between PA and neuroanatomical volume, noting that higher levels of PA may help to prevent age-related declines in hippocampal and cerebral volumes [53]. This might partially explain the observed association between lack of PA and cognitive impairment in older adults in the present study. Evidence from India also supports these associations. A study showed that regular PA among Indian adults was linked to better memory retention and cognitive performance [54]. Consistent with these findings Sekhar and Muhammad (2023) reported that yoga or meditation showed the strongest independent association with cognitive function among elderly [55]. Similarly, Gupta and Kumar (2024) found that older Indian adults who engaged in regular walking or yoga exhibited lower levels of cognitive complaints compared to sedentary individuals [56].

Our study displayed that there is a significant link among poor PAs and greater depressive symptoms during old age, which is in line with the previous studies [57–59]. Moreover, Han et al. (2021) discovered that higher levels of depressive symptoms related with the higher likelihood of cognitive impairment in a 10-year follow-up period in a population-based study [60]. Lindwall et al. (2012) found that older women who were more physically active, particularly those who walked more, showed less cognitive decline over 8 years [61]. In a review-based study, Mahindru et al. (2023) found comparable evidence linking physical inactivity with elevated depressive symptoms among older adults in India [62]. Wassink-Vossen et al. (2014) reported that older individuals experiencing depressive symptoms engaged in lower levels of PA compared with those without depressive symptoms [63]. They also noted that depressed older individuals who were physically inactive experienced increased functional impairments. Zhang et al. (2021) examined older persons with and without depressive symptoms utilizing a standardized depression scale and found that those exhibiting depressive symptoms engaged in significantly lower levels of physical activity [64]. A considerable body of research supports the connection between increased engagement in PA and reduced depressive symptoms and mental health issues globally and India [62, 65–69].

Beyond the hypothesized associations of PA with depressive symptoms and cognitive impairment, the study highlights that extreme sedentary behaviour was more common among those with lower or middle socioeconomic status and elevated educational qualifications. The results reveal that people with higher educational attainment and from lower and middle socioeconomic strata may still opt for less healthy lifestyles, which is consistent with earlier research by Brandao et al. (2011) [70]. We hypothesized that sedentary behaviour among well-educated individuals in lower- and middle-income brackets may be driven by limited time for regular exercise due to the pressures of globalization and numerous daily responsibilities [71]. Our study observes that individuals from lower and middle socioeconomic backgrounds exhibit more cognitive impairment and depressive symptoms than their wealthier counterparts, which may explain their lack of time for regular physical activities. Freeman et al. (2016) report that depressive symptoms are more common among those with lower socioeconomic resources and impaired social, health, and functional functioning [72]. Indian context where joint family structures have traditionally served as protective factors for older adults. A study by Tiwari (2020) in India found that elderly individuals residing in joint families had better cognitive health and lower depressive symptoms than those in nuclear or single-person households [73]. Similarly, being currently employed was allied with a lower likelihood of cognitive impairment and depressive symptoms, possibly due to continued engagement, sense of purpose, and routine associated with working life. Retired or never-worked individuals, in contrast, showed higher risks, supporting prior findings that social inactivity and loss of occupational identity can negatively affect mental well-being in older age [74, 75]. Further, the findings suggest that enhancing PA levels in older people might lead to improved cognitive abilities and potentially reduce symptoms of depression. For example, a six-month program of moderate exercise, consisting of 150 min weekly (or 50 min thrice a week) of activities like walking or gentle strength training, was found to boost cognitive performance in older adults [76].

The findings show that both vigorous and holistic physical activities have especially strong protective associations for the oldest-old. Even engaging in any one type of PA was associated with a noticeable drop in depressive symptoms and cognitive impairment among individuals. This pattern aligns with prior research suggesting that PA becomes increasingly associated with better psychological well-being and cognitive health in advanced age, possibly due to its role in preserving functional independence, enhancing social engagement, and improving neuroplasticity [24, 62, 77]. Older adults who remain physically active often experience enhanced self-efficacy and social interaction which can buffer against depressive mood and cognitive decline [64, 78, 79]. Furthermore, the especially close correlation between holistic activities like yoga, meditation, and stretching and fewer depressive symptoms and cognitive impairment among all age groups supports the accumulating evidence in India and other low- and middle-income settings [55, 80]. These activities integrate physical movement with mindfulness and relaxation, potentially exerting dual physiological and psychological advantages through stress reduction, improved sleep, and better emotional regulation [81]. Our findings add that this association extends to depressive symptoms and to younger age cohorts underscoring the value of holistic activities as a broad non-pharmacological intervention across the adult lifespan. In addition, broader evidence highlights that sustained engagement in PA contributes to improved overall well-being including reduced stress and enhanced productivity even beyond older population [82].

In summary, the findings of this research suggest that non-engagement in PA is associated with a higher likelihood of depressive symptoms and cognitive decline, in contrast to individuals who maintain a routine of daily exercise. Moreover, adults from lower- and middle-socioeconomic groups appear particularly susceptible to worsening depressive symptoms and cognitive functioning. These results highlight the potential of PA as a non-pharmacological strategy to support the management of depressive symptoms and cognitive health, as it not only alleviates distressing symptoms but also facilitates positive behavioural adaptations, encourages healthier lifestyle practices, and contributes to an overall enhancement of quality of life. Integrating regular PA into public health interventions may therefore serve as a valuable approach to mitigate the burden of depressive symptoms and cognitive deterioration in vulnerable populations.

### Strengths and limitations of the study

To our knowledge, there are currently only a few studies exploring the association between various types of PA and the incidence of cognitive deterioration and depressive symptoms in the older population, despite these factors having substantial effects on health outcomes and quality of life. Our research addresses this knowledge gap by investigating the connection between participation in physical activities and the chances of experiencing cognitive decline and depressive symptoms. We conduct this study on a nationwide scale, leveraging the latest information from the comprehensive LASI survey. The incorporation of clinical data, in addition to survey responses, strengthens the validity and reliability of our findings.

The present study has some limitations that require acknowledgment. Firstly, this study is based on a cross-sectional design, which prevents us from determining causal effect relationships between interpreters of interest (i.e., physical activity) and outcomes (i.e., depressive symptoms and cognitive function). It is also possible that individuals experiencing depressive symptoms or cognitive decline were less likely to engage in PA, rather than low activity levels leading to these conditions. Secondly, our study relied on self-reported data for PA and depressive symptoms, collected through questionnaires that focused on the experiences of participants in the week prior to assessment. This approach yielded subjective measurements of both depressive symptoms severity and PA engagement. Therefore, these self-assessment tools require additional investigation to evaluate potential recall bias, especially among participants particularly in older age groups. Thirdly, the study did not account for variations in BMI categorization between adults and older adults. As BMI cut-offs may not accurately capture the true nutritional status of older individuals, this remains an important limitation to consider in interpreting the findings. Lastly, by excluding the cases with incomplete data from the full-case analysis, we may have introduced a source of probable bias in our results. To gain a more profound insight into these relationships, longitudinal studies will be needed in the future to determine the temporal associations and pathways of causation as well as the long-term effects of sustained involvement in various types of physical activities with cognitive functioning and depressive symptoms.

## Conclusion

This nationwide study provides significant evidence of the relationships among PAs and depressive symptoms and cognitive ability among Indian older adults. Our results reveal that engagement in various forms of PA, including moderate, vigorous, and holistic well-being activities, is associated to lower occurrence of cognitive impairment and depressive symptoms. This study highlights the importance of physical exercise as a therapeutic intervention (non-drug) that can be applied to promote cognitive performance and mental health in ageing populations. Age, socioeconomic status, educational attainment, marital status and living arrangements were also were observed to play a substantial role in the prevalence of cognitive function decline and depressive symptoms. However, the study is cross-sectional, and the findings cannot be used to draw causal conclusions; they demonstrate the significance of physical activity promotion among older adults as a component of public health policy. The findings suggest that targeted interventions to increase PA levels, especially among lower socioeconomic groups and those with less education, could potentially improve mental health outcomes in this population. Longitudinal studies are also required to present causal pathways and to determine the long-term effect of various levels of PAs and modalities on depressive symptoms and cognitive functioning during old age.

## Data Availability

The study is based on a secondary data source that is publicly accessible and freely available through [https://g2aging.org/](https:/g2aging.org) .

## Abbreviations

BMI: Body Mass Index
CI: Confidence Interval
CES-D: Center for Epidemiological Studies-Depression
CoI: Cognitive Impairment
IIPS: International Institute for Population Sciences
LASI: Longitudinal Ageing Study in India
NCoI: No Cognitive Impairment
PA: Physical Activity
PR: Prevalence Ratio
WHO: World Health Organization
